# The Cx43-Mediated Autophagy Mechanism Influences Triple-Negative Breast Cancer Through the Regulation of Rab31

**DOI:** 10.3390/cancers17243923

**Published:** 2025-12-08

**Authors:** Jiao Yang, Die Wu, Ting Yang, Zi-Jing Lin, Pei-Yao Jiang, Ting-Rui Wang, Zheng-Jia Lu, Lu Wang, Jia Ming

**Affiliations:** 1 Department of Breast and Thyroid Surgery, The Second Affiliated Hospital of Chongqing Medical University, Chongqing 400010, China; 2 Da-Ping Hospital, Army Medical University, Chongqing 400042, China

**Keywords:** breast cancer, TNBC, Cx43, Rab31, autophagy

## Abstract

**Background:** Connexin43 (Cx43) is recognized as a transmembrane protein; its precise expression profile and molecular mechanisms in triple-negative breast cancer (TNBC) remain unclear. **Methods:** We systematically analyzed Cx43 expression in over 60 breast cancer cell lines from the CCLE and HPA databases. Immunohistochemical evaluation compared Cx43 expression between TNBC tissues and adjacent normal tissues. Cx43 expression was assessed in normal breast epithelial cells (MCF-10A) and two TNBC cell lines (MDA-MB-231 and BT-549) using qRT-PCR and Western blot. Functional assays (CCK8, wound healing, transwell) evaluated TNBC progression following Cx43 interference or overexpression. Rab31, a Cx43-interacting protein, was identified via bioinformatics, immunofluorescence, and Co-IP. Autophagy-related proteins (ULK1, ATG5, LC3, and p62) were analyzed after Cx43 or Rab31 modulation. Finally, a nude mouse model validated Cx43’s in vivo effects on tumor growth and associated molecular changes. **Results:** Cx43 was upregulated in TNBC tissues and cell lines. Overexpression enhanced proliferation, migration, and invasion, while knockdown suppressed these effects. Cx43 co-expressed with Rab31, regulating its protein levels and autophagy. Rab31 interference reversed Cx43-mediated autophagy and oncogenic behaviors. In vivo, Cx43 promoted tumor growth and modulated Rab31/autophagy pathways. **Conclusions:** The Cx43/Rab31 axis drives autophagy to facilitate TNBC progression, highlighting Cx43 as a potential therapeutic target. Our findings provide mechanistic insights for improving TNBC treatment.

## Simple Summary

This study investigates the novel mechanism by which Connexin 43 (Cx43) promotes triple-negative breast cancer (TNBC) progression by regulating Rab31-mediated autophagy. We demonstrated that Cx43 is significantly upregulated in TNBC tissues and cell lines. Functional assays confirmed that Cx43 overexpression enhances cell proliferation, migration, and invasion capabilities. Mechanistically, Cx43 physically interacts with Rab31 and positively regulates its expression, subsequently activating the ULK1-mediated autophagy pathway, as evidenced by increased LC3-II conversion and p62 degradation. In vivo experiments further validated that Cx43 overexpression promotes tumor growth and upregulates Rab31 and autophagy-related proteins. Our study reveals for the first time the pro-tumorigenic role of the “Cx43-Rab31-autophagy” axis in TNBC, providing a potential therapeutic target for TNBC treatment.

## 1. Background

Breast cancer is a prevalent and highly heterogeneous malignant tumor among women. According to the latest data from the International Agency for Research on Cancer (IARC) in 2022, breast cancer accounts for approximately 24% of all female cancers globally, making it the most common malignant tumor in women [1]. The incidence rate has been rising annually, yet the mortality rate has not shown a significant decline. Particularly, triple-negative breast cancer (TNBC), characterized by the absence of estrogen receptors (ERs), progesterone receptors (PRs), and human epidermal growth factor (HER2) expression, is the most aggressive subtype of breast cancer. It exhibits the highest metastatic potential and poorest prognosis, with a 5-year survival rate of approximately 70% [2], which is significantly lower than the over 90% 5-year overall survival rate observed in other subtypes of breast cancer [3]. Consequently, the prevention and treatment of triple-negative breast cancer warrant greater attention and research efforts.

Connexins (Cxs) represent a family of transmembrane proteins that form gap junction (GJ) channels, facilitating intercellular communication between adjacent cells. Connexin43 (Cx43), one of the most extensively studied and highly expressed gap junction proteins [4], exhibits abnormal expression patterns in various types of tumors, including liver, prostate, and breast cancers [5,6,7]. According to current research, Cx43, as a transmembrane protein, demonstrates abnormal expression associated with cancer phenotypes and plays a dual role in tumor progression—both inhibiting and promoting tumorigenesis. However, there is no definitive consensus on whether Cx43 acts as an inhibitor or promoter in breast cancer studies. Our previous findings revealed that Cx43 expression was detectable in both surgically resected breast cancer specimens and metastatic lymph nodes, with significantly elevated expression observed in metastatic lymph nodes [8]. This suggests that Cx43 may assume distinct roles during breast cancer progression, warranting further investigation into its underlying mechanisms. Additionally, prior studies have demonstrated that Cx43 expression is detectable in circulating tumor cells (CTCs), and its expression may correlate with high Ki67 expression, tumor diameter exceeding 2 cm, and lymph node metastasis [9]. Given that CTCs serve as precursors for distant metastasis, the presence of Cx43 in CTCs indicates its potential involvement as an oncogenic factor in the distant metastasis of breast cancer.

Autophagy is a critical intracellular degradation process that eliminates misfolded proteins and damaged organelles, thereby maintaining cellular homeostasis [10]. In recent years, research has demonstrated that autophagy plays a complex and context-dependent role in tumor initiation and progression [11,12], which is influenced by tumor stage, specific oncogenic mutations, and cellular microenvironmental factors. Under physiological conditions, low-level autophagy removes metabolic waste, preserves genomic stability, and suppresses tumorigenesis [13]. However, in established tumors, autophagy enables cancer cells to withstand various stress responses and provides metabolic substrates via intracellular recycling, thereby supporting energy metabolism and promoting tumor progression [14,15,16]. Emerging evidence has begun to delineate a functional interplay between Cx43 and the autophagic process in cancer. On one hand, Cx43 can regulate autophagic activity. For instance, Cx43 has been shown to induce protective autophagy in glioma cells under temozolomide chemotherapy, thereby promoting cell survival and treatment resistance [17]. On the other hand, autophagy serves as a key degradation pathway for Cx43. The degradation of Cx43 via autophagy has been documented to impair gap junctional intercellular communication in the tumor microenvironment [18], This reciprocal regulation between Cx43 and autophagy introduces a compelling layer of complexity to their respective roles in cancer biology. The involvement of Cx43 in triple-negative breast cancer through autophagy-mediated mechanisms remains underexplored in the literature.

GeneMANIA and STRING interactive platforms were utilized to predict the potential mechanisms involved in breast cancer metastasis, specifically focusing on the possible target molecules that interact with Cx43. The results indicated that Ras-related protein Rab-31 (Rab31) might bind to Cx43. Both proteins exhibit similar expression patterns and cellular localization. Rab31, a member of the Rab family of small GTPases, is believed to play a critical role in cancer initiation and progression [19]. Rab proteins are known to regulate autophagy by mediating autophagosome formation, autophagosome-endosome fusion, and autophagosome-lysosome fusion [20], as well as participating in various biological processes such as membrane trafficking, signal transduction, and cell proliferation [21]. Studies have demonstrated that Rab31 is highly expressed in multiple cancers [22,23,24], and its expression levels influence tumor invasion, metastasis, proliferation, and other phenotypic characteristics through the activation of different signaling pathways, including transforming growth factor β (TGF-β)-mediated autophagy and the PI3K/Akt signaling pathway [25]. These malignant phenotypes serve as key prerequisites for rapid tumor growth. As an essential regulator of vesicle and substance transport within the cytoplasm, Rab31 may promote the epithelial–mesenchymal transition (EMT) of tumor cells via autophagy, thereby affecting breast cancer progression [26,27]. Consequently, we hypothesize that Cx43 may interact with Rab31 to contribute to the progression of triple-negative breast cancer through autophagy. The objective of this study is to explore whether the Cx43/Rab31 axis influences the biological characteristics and distant metastasis of triple-negative breast cancer via the autophagy signaling pathway, providing additional insights and experimental evidence for breast cancer treatment.

## 2. Materials and Methods

### 2.1. Clinical Breast Cancer Tissue Samples

All breast cancer and paired adjacent tissues were collected from five patients who underwent surgical resection at the Second Affiliated Hospital of Chongqing Medical University in 2024. The inclusion criteria comprised patients with an initial diagnosis of TNBC, no prior neoadjuvant therapy, and pathological confirmation of TNBC. Exclusion criteria involved patients with other concurrent malignancies or those who had received preoperative radiotherapy, chemotherapy, or endocrine therapy. Immediately following surgical resection, the samples were fixed and preserved in 4% paraformaldehyde solution. Written informed consent was obtained from all patients.

### 2.2. Cell Lines and Culture Conditions

BT-549, MDA-MB-231, and MCF-10A cells were obtained from ATCC (Manassas, VA, USA). MDA-MB-231 cells were cultured in DMEM (Gibco, Thermo Fisher Scientific, Waltham, MA, USA), while BT-549 cells were cultured in RPMI-1640 (Gibco, Thermo Fisher Scientific, Waltham, MA, USA), both supplemented with 10% FBS and 1% penicillin-streptomycin. MCF-10A cells were cultured in a specialized medium (The basic ingredients were DMEM/F12 (1:1), supplemented with 5% horse serum, 20 ng/mL EGF, 0.5 mg/mL hydrocortisone, 100 ng/mL cholera toxin, 10 μg/mL insulin, and 1% penicillin/streptomycin). Cell subculturing was performed by enzymatic dissociation with trypsin-EDTA (0.25%) (Beyoutime, Shanghai, China). All experiments were conducted with cells at passage numbers not exceeding 20. All cultures were maintained at 37 °C with 5% CO_2_ in humidified incubators. Mycoplasma testing using the Beyotime detection kit (Beyoutime, Shanghai, China) yielded negative results.

### 2.3. siRNA Transfection, Lentivirus Infection, and Overexpression Constructs

Three siRNAs (si1-Cx43, si2-Cx43, si3-Cx43, NO. RX078575) and a negative control (siRNA-NC) were designed to target Cx43 expression. A lentiviral vector overexpressing Cx43 (OE-Cx43) and a control (NC) were purchased from Jiangsu KeyGEN Biotechnology Co., Ltd. (Nanjing, China) Stable Cx43-overexpressing cell lines were established via puromycin selection. Three siRNAs targeting Rab31 (si1-Rab31, si2-Rab31, si3-Rab31, NO. RX088541) and negative control (siRNA-NC), overexpression plasmid for Rab31 (OE-Rab31), and its empty vector control (Vector) were synthesized from KeyGEN Biotechnology Co., Ltd. (Nanjing, China) All transfections were performed using Lipo8000 reagent (Beyotime, Shanghai, China) following the manufacturer’s protocol. All the primer sequences were listed in Table 1.

### 2.4. qRT-PCR

Total RNA was extracted using the RNA extraction kit (Agbio, Wuhan, China). And then transcribed into cDNA using PrimeScript^TM^ RT reagent Kit with gDNA Eraser (Takara, Chengdu, China). Quantitative real-time PCR was performed using TB Green *Premix Ex Taq* II (Takara, Chnegdu, China) on a CFX96 real-time PCR System. All oligonucleotide primers were obtained from Agbio, China. GAPDH was used as a control. The program was: 95 °C for 30 s, followed by 40 cycles of 95 °C for 5 s and 60 °C for 30 s, with a final extension at 65 °C for 5 s and 95 °C for 10 s. Triplicate wells were used for each sample, and relative mRNA expression levels were calculated using the 2^−ΔΔCT^.

### 2.5. Western Blot (WB)

Cells were lysed with RIPA buffer containing phosphatase and protease inhibitors (100:1:1). Lysates were centrifuged at 12,000 rpm for 15 min at 4 °C, and the supernatant was collected. Protein concentration was measured using the BCA method. Equal amounts of protein (30 µg) were loaded into each well and separated by SDS-PAGE under constant voltage. Proteins were transferred to PVDF membranes at 250 mA on ice. Membranes were blocked with 5% skim milk for 1 h at room temperature, washed three times with TBST for 10 min each, and incubated overnight at 4 °C with primary antibodies against Cx43 (CST #3512, 1:1000), Rab31 (proteintech #15485-1-AP, 1:2000), GAPDH (proteintech #10494-1-AP, 1:10,000), p62 (Abmart #T55546, 1:8000), LC3B((Abmart #T55992, 1:2000), ULK1(Abmart #T56902, 1:2000), and ATG5 (Abmart #T55766, 1:2000). After washing with TBST three times for 10 min each, membranes were incubated with secondary antibodies for 1 h at room temperature. Washing three times with TBST, the membranes were exposed to a chemiluminescent substrate, and protein bands were visualized. Relative protein expression levels were quantified using ImageJ (version 1.54).

### 2.6. Transwell

Migration: Cells from each group were resuspended in serum-free medium at 2.5 × 10^5^ cells/mL. A 200 μL aliquot was added to the upper chamber, and 600 μL of medium with 10% FBS was added to the lower chamber. Chambers were incubated 24 h under standard conditions. The chambers were removed, fixed with 4% paraformaldehyde, and stained with crystal violet. After washing with PBS, the remaining cells in the upper chamber were removed with a cotton swab. Following air drying, 3–5 random fields were imaged and counted under a microscope.

Invasion: Matrigel was diluted 1:6 in serum-free medium on ice. Transwell chambers were placed in 24-well plates. A 60 μL volume of diluted Matrigel was added to the upper chamber and solidified at 37 °C for 4 h in the incubator.

For transwell assays, Corning model 3422 (Corning, New York, NY, USA) (8 μm pore size, 6.5 mm diameter) was used for migration assays, and Matrigel (Corning) was used for invasion assays. All the data were analyzed using ImageJ.

### 2.7. Wound Healing

On the day of the experiment, a scratch was made with a 200 μL pipette tip perpendicular to the horizontal line on the bottom of the six-well plate. Detached cells were removed by washing with PBS, and 2 mL of serum-free medium was added to each well. The initial scratch width was recorded at 0 h using an inverted microscope. Scratch widths were measured again at 24 and 48 h. Relative migration rates were calculated using the formula: relative migration rate = (initial distance-distance at 24 h or 48 h)/initial distance.

### 2.8. CCK-8

The transfected cells were divided into negative control and treatment group. After a 24 h transfection, cells were harvested by trypsinization and centrifuged, the density was adjusted at 2 × 10^4^ cells/mL, and we inoculated 100 μL/well into a 96-well plate. After cell adherence (0 h time point), 10 μL of CCK-8 solution was added to each well at 24, 48, 72, and 96 h. Absorbance was measured at 450 nm after 2 h of dark incubation, and the data were used for a growth curve.

### 2.9. Immunofluorescence

Cells were fixed with 4% paraformaldehyde for 30 min, permeabilized with 0.1% Triton X-100 for 15 min, and blocked with 10% goat serum for 30 min at room temperature. The primary antibody against Cx43 (1:200) was incubated overnight at 4 °C. Cells were washed three times with PBS, followed by incubation with the secondary antibody for 1 h at room temperature. After washing, cells were stained with green fluorescent dye for 10 min and washed again three times with PBS. The primary and secondary antibodies were eluted in a weakly alkaline buffer for 30 min at room temperature. Cells were re-blocked and incubated with the primary antibody against Rab31 (1:100) overnight at 4 °C, followed by staining with red fluorescent dye. DAPI staining was performed in the dark at room temperature for 10 min. Slides were sealed with an anti-fade mounting medium and observed under a confocal microscope.

### 2.10. Co-IP

After centrifugation, the protein supernatant was divided into three groups: Input, IgG, and IP groups. For the Input group, loading buffer was added, followed by boiling for 10 min and storage at −80 °C. For the IgG and IP groups, 5 µg of antibody was mixed with protein A/G magnetic beads (MCE) and rotated at 4 °C for 2 h to form the bead-antibody complex (Cx43 (CST#3512, 1:400), Rab31 (proteintech#15485-1-AP, 1:200), SDS-PAGE (Servicebio#G2075), IgG (biyotime#A7016), ProteinA/G Magnetic Beads (MCE#HY-K0202)). The protein supernatant was added and rotated at 4 ° C overnight to form bead–antibody–antigen complexes. The next day, the complexes were washed again with PBST and separated on a magnetic rack. A total of 50 µL of 1× SDS-PAGE Loading Buffer was added, and the mixture was boiled for 10 min. The beads were separated, and the supernatant was collected and combined with the Input group samples for Western blot.

### 2.11. Immunohistochemistry (IHC)

Tissues were fixed in 4% paraformaldehyde and embedded in paraffin for sectioning. Paraffin sections were deparaffinized, rehydrated, and subjected to antigen retrieval using EDTA solution in a microwave oven. Sections were permeabilized with 0.3% Triton X-100 for 10 min, followed by blocking of endogenous peroxidase activity for 30 min and incubation with goat serum for 1 h. Primary antibodies were applied overnight at 4 °C. After washing with PBS, a secondary antibody was applied at 37 °C for 30 min, followed by DAB staining under microscopic observation. Sections were counterstained with hematoxylin, dehydrated, cleared with xylene, mounted, examined microscopically, and photographed. The immunohistochemical staining results were independently evaluated by two pathologists who were blinded to the clinical data. A semi-quantitative scoring method was applied, which incorporated both staining intensity (0 = negative, 1 = weak, 2 = moderate, 3 = strong) and the proportion of positive cells (1 = 0–25%, 2 = 26–50%, 3 = 51–75%, 4 = >75%). The final immunoreactivity score was calculated by multiplying the intensity and percentage scores, yielding a total score ranging from 0 to 12. Scores of 0–3 were defined as low expression, 4–7 as moderate expression, and >7 as high expression.

### 2.12. In Vivo Tumorigenesis Assay

MDA-MB-231 cells stably transfected with Cx43 overexpression lentivirus or control vector were trypsinized, centrifuged at 1000 rpm for 5 min, and resuspended in PBS. The cell suspension (1 × 10^7^ cells/100 μL) was inoculated into the right mammary fat pad of 4-week-old female BALB/c nude mice. The mice were randomly assigned to experimental groups (*n* = 6 per group). And were housed under specific pathogen-free (SPF) conditions with a controlled 12/12 h light/dark cycle, temperature (22 ± 2 °C), and humidity (50 ± 10%), and were allowed access to food and water ad libitum. Tumor volumes were measured every three days using digital calipers with the formula: tumor volume = (length × width^2^)/2. Tumor-bearing mice were humanely euthanized prior to the scheduled endpoint if any of the following conditions occurred: the tumor volume exceeded 2000 mm^3^, the tumor exhibited ulceration, the animal showed signs of significant distress, or the weight loss exceeded 20%. After 35 days, animals were euthanized, and tumors were excised for final volume and weight measurements.

### 2.13. Statistical Analysis

Statistical analyses were performed using GraphPad Prism 9.0. Continuous data are presented as mean ± SD. In vitro experiments, WB and qRT-PCR were repeated 3 times (both biological replicates and technical replicates). Phenotypic experiments were repeated 3 times (technical replicates), while in vivo experiments used 6 mice per group. Two-group comparisons used unpaired t-tests, while multi-group comparisons used one-way ANOVA with Tukey’s post hoc test. A *p*-value < 0.05 was considered significant.

### 2.14. Bioinformatics and Database Analyses

Cx43 expression in breast cancer cell lines using data downloaded from the Cancer Cell Line Encyclopedia (CCLE) (public 23Q2), supplemented by parallel data extraction from the Human Protein Atlas (HPA). The breast cancer cell lines were stratified into Cx43-high and Cx43-low groups based on whether their Cx43 expression level was above or below the mean expression across all lines.

## 3. Results

### 3.1. Cx43 Was Upregulated in Triple-Negative Breast Cancer Tissues and Cell Lines

**Figure 1:**
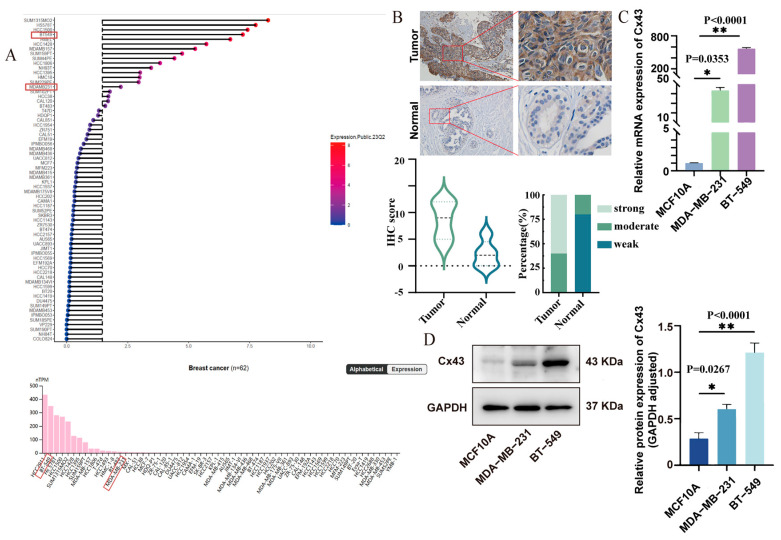
Cx43 is upregulated in triple-negative breast cancer (TNBC) tissues and cells. (**A**) Cx43 expression in breast cancer cell lines (CCLE and HPA data). (**B**) Representative IHC photomicrographs and quantitative analysis of Cx43 expression in TNBC tumor tissues and adjacent normal tissues (n = 5). (**C**) qRT-PCR and (**D**) WB were utilized to assess Cx43 expression levels (n = 3 independent experiments (biological replicates). Statistical significance was determined using one-way ANOVA with Tukey’s post hoc test. Mean ± SD, * *p *< 0.05, ** *p *< 0.01). Original western blots are presented in File S1.

To study Cx43 expression in breast cancer, we analyzed data from over 60 cell lines in the CCLE and HPA databases. Results showed that more than 70% of high Cx43-expressing cell lines were TNBC, while other subtypes had minimal or low expression (Figure 1A). We also examined TNBC tissue samples from our hospital using immunohistochemistry, showing an upregulation trend of Cx43 expression in tumor tissues compared to normal adjacent tissues (Figure 1B). Additionally, we observed significantly higher levels of both Cx43 mRNA (Figure 1C) and protein (Figure 1D) in MDA-MB-231 and BT-549 cells compared to the MCF10A. These findings aligned with the database analysis. We then selected Cx43 for further investigation, using overexpression in MDA-MB-231 and knockdown in BT-549 cells.

### 3.2. Validation of the Efficiency of Cx43 Interference and Overexpression

**Figure 2:**
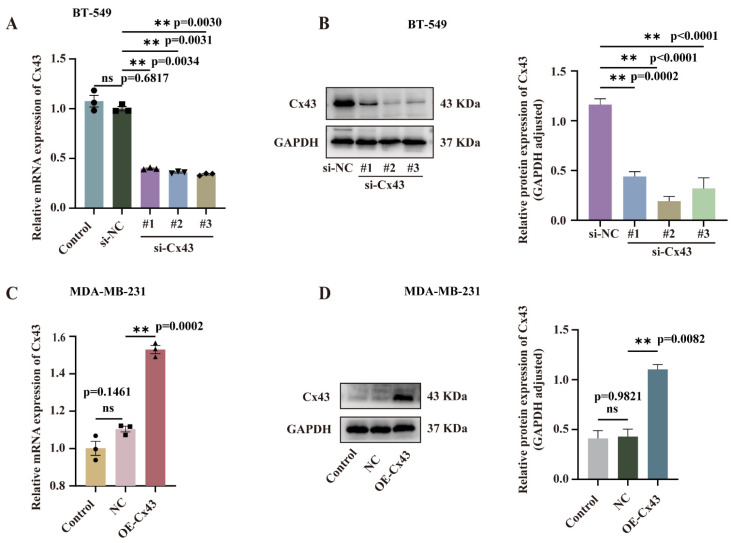
Validation of Cx43 expression efficiency. (**A**) qRT-PCR and (**B**) WB were used to assess the interference efficiency of Cx43. (**C**) qRT-PCR and (**D**) WB were used to detect the overexpression efficiency of Cx43. (n = 3 independent experiments (biological replicates). Statistical significance was determined using one-way ANOVA with Tukey’s post hoc test. Mean ± SD, ** *p* < 0.01, ns, not significant (*p *> 0.05)). Original western blots are presented in File S1.

To study the impact of Cx43 on triple-negative breast cancer cells, three siRNA fragments targeting Cx43 (si-Cx43) and negative control (si-NC) were designed. BT-549 cells transfected with these siRNAs showed significantly reduced Cx43 mRNA and protein levels compared to si-NC groups, as measured using qRT-PCR and Western blotting (Figure 2A,B). The most efficient si-Cx43 fragment was chosen for further studies. Additionally, MDA-MB-231 cells were transfected with a lentivirus overexpressing Cx43 (OE-Cx43) and a control (NC). A stable cell line was established via puromycin selection, and Cx43 expression was confirmed by qRT-PCR and Western blotting (Figure 2C,D), showing significantly higher Cx43 levels in OE-Cx43 cells than NC.

### 3.3. Inference of Cx43 Expression Significantly Suppressed Cell Proliferation, Migration, and Invasion, Whereas the Overexpression of Cx43 Promoted These Processes

**Figure 3:**
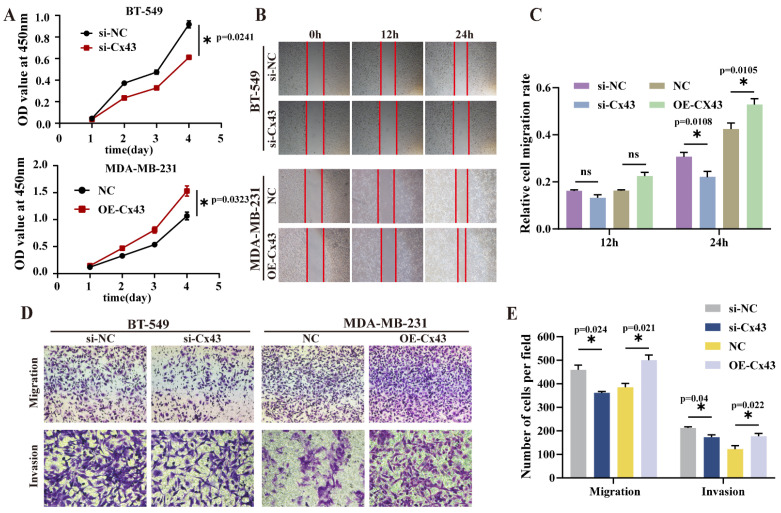
Effects of Cx43 on the malignant phenotypes of triple-negative breast cancer. (**A**) The CCK8 assay was employed to evaluate the proliferative activity (*statistical significance was determined using two-way ANOVA*). (**B**,**C**) Wound healing assay was employed to evaluate cell migration and migration rate; (**D**,**E**) transwell assay was employed to evaluate cell migration and invasion ((n = 3, *technical replicates). Statistical significance was determined using unpaired t-test (two-group comparison)*, mean ± SD, * *p* < 0.05, ns, not significant (*p *> 0.05)).

We used CCK-8 assays to evaluate the proliferative capacity. Results showed that inhibiting Cx43 reduced BT-549 cell proliferation, while Cx43 overexpression increased MDA-MB-231 cell proliferation (Figure 3A). Wound healing assays revealed lower BT-549 cell mobility with Cx43 knockdown and higher MDA-MB-231 cell migration with Cx43 overexpression (Figure 3B,C). Transwell migration assays showed fewer BT-549 cells passing through the membrane after Cx43 knockdown and more MDA-MB-231 cells with Cx43 overexpression than control. Similarly, transwell invasion assays demonstrated reduced BT-549 cell invasion and increased MDA-MB-231 cell invasion with Cx43 overexpression (Figure 3D,E). These findings suggest that inhibiting Cx43 suppresses BT-549 cell migration and invasion, while Cx43 overexpression enhances these abilities in MDA-MB-231 cells.

### 3.4. Cx43 Interacts with Rab31 to Regulate the Autophagy Pathway in Triple-Negative Breast Cancer Cells

**Figure 4:**
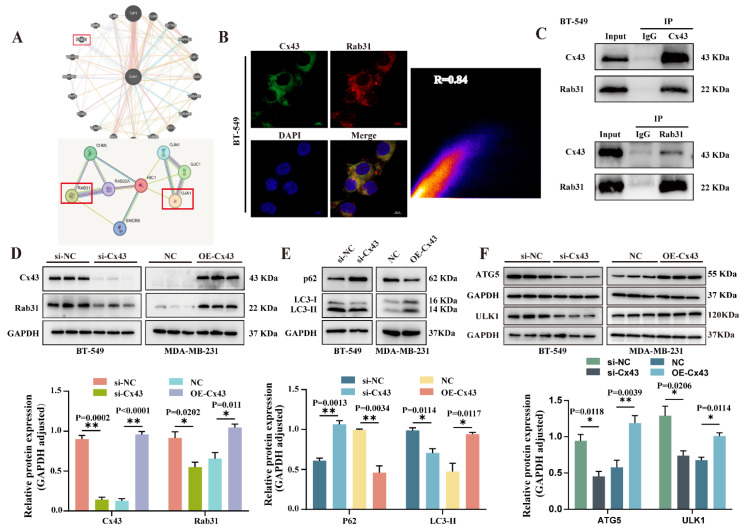
Interaction between Cx43 and Rab31 and its functional role in autophagy. (**A**) The GeneMANIA and STRING databases identify Rab31 as a potential Cx43-interacting protein. (**B**) Confocal microscopy shows co-localization of Cx43 and Rab31 in BT-549 (scale bar = 10 μm). (**C**) The interaction between Cx43 and Rab31 was confirmed using co-immunoprecipitation (Co-IP). (**D**) WB analysis of Rab31 expression after modulation of Cx43 levels. (**E**,**F**) WB evaluates the impact of autophagy by Interference and overexpression of Cx43 (n = 3 independent experiments (biological replicates). Statistical significance was determined using unpaired *t*-test. Mean ± SD, * *p* < 0.05, ** *p* < 0.01). Original western blots are presented in File S1.

To investigate the molecular mechanisms of Cx43 in triple-negative breast cancer, we performed bioinformatics analyses using GeneMANIA and STRING databases. These analyses predicted that Cx43 may interact with various proteins, including other connexins such as Cx32 and Cx26, cytoskeletal protein TUBB, and proteins involved in the MAPK, Rab31, and PI3K/AKT signaling pathways (Figure 4A). Among them, Rab31 plays a key regulatory role in vesicle and membrane trafficking. We validated the interaction between Cx43 and Rab31 using immunofluorescence (IF) and co-immunoprecipitation (Co-IP). The results confirmed that Cx43 and Rab31 co-localize and interact within the cytoplasm (Figure 4B,C). Given Rab31’s role in vesicle trafficking, we examined its expression levels after Cx43 knockdown and overexpression. Western blot confirmed that Rab31 expression decreased upon Cx43 knockdown and increased upon Cx43 overexpression (Figure 4D). The aforementioned studies indicate that Cx43 may influence the progression of triple-negative breast cancer via Rab31-mediated mechanisms.

Previous studies suggested Rab31 may influence tumor progression via autophagy. Here, we analyzed the expression of autophagy-related proteins p62 and LC3 by Western blot. Cx43 interference in BT-549 cells decreased LC3 expression and p62 was increased, while Cx43 overexpression in MDA-MB-231 cells increased LC3 expression and p62 expression was significantly reduced (Figure 4E and Figure S1). We also examined ULK1 and ATG5, finding their levels decreased with Cx43 interference but increased with Cx43 overexpression in MDA-MB-231 cells (Figure 4F). The change in autophagy markers may indicate that Cx43 expression promotes or inhibits autophagy.

### 3.5. Rab31 Was Highly Expressed in Triple-Negative Breast Cancer Cells, and Interference of Rab31 Expression Inhibited Cell Proliferation, Migration, and Invasion, While Overexpression of Rab31 Promoted These Processes

**Figure 5:**
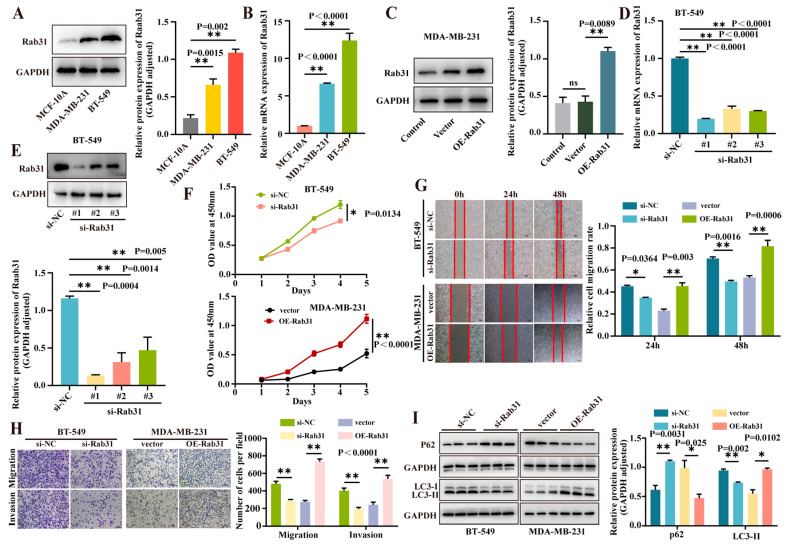
Functional characterization of Rab31 in TNBC. (**A**) WB and (**B**) qRT-PCR were utilized to evaluate the differential expression of Rab31 at the protein and mRNA levels in normal breast epithelial cells (MCF-10A) and triple-negative breast cancer cells (MDA-MB-231, BT-549). (**C**) WB analysis confirming Rab31 overexpression efficiency. (**D**) qRT-PCR and (**E**) WB were used to assess the interference efficiency of Rab31. (**F**) A CCK8 assay was utilized to measure cell proliferation following Rab31 modulation (statistical significance was determined using two-way ANOVA). (**G**) A wound healing assay assesses cell migration. (**H**) A transwell assay was used to evaluate cell migration and invasion ability. (**I**) The influence of Rab31 on autophagy-related protein expression was examined using Western blotting (n = 3 independent experiments (biological replicates). Two-group comparisons used unpaired *t*-tests, while multi-group comparisons used one-way ANOVA with Tukey’s post hoc test. Mean ± SD, * *p* < 0.05, ** *p* < 0.01). Original western blots are presented in File S1.

To determine if Rab31 is differentially expressed in triple-negative breast cancer, we used qPCR and Western blot analyses to detect its mRNA and protein levels in normal epithelial cells (MCF10A) and triple-negative breast cancer cell lines (MDA-MB-231 and BT-549). Rab31 was significantly upregulated in both cancer cell lines (Figure 5A,B). These findings align with the Cx43 expression trend in triple-negative breast cancer cells. To explore the mechanisms involving Cx43, Rab31, and autophagy, as well as Rab31’s impact on TNBC cells, we conducted a series of experiments. MDA-MB-231 cells (low Rab31) were transfected with a Rab31 overexpression plasmid, while BT-549 cells (high Rab31) were transfected with siRNA targeting Rab31. We designed three si-Rab31 fragments and their control (si-NC), along with Rab31 overexpression plasmids and control (vector). Rab31 mRNA and protein levels were measured using qPCR and Western blotting. The results showed MDA-MB-231 cells were transfected with an OE-Rab31 plasmid or vector, and Western blotting confirmed significantly increased Rab31 expression in OE-Rab31-transfected cells (Figure 5C). And it significantly reduced Rab31 expression in the si-Rab31 group compared to si-NC (Figure 5D,E). The most efficient si-Rab31 sequence was selected for further assays.

We investigated the role of Rab31 in TNBC cells. CCK8 assays showed that interfering with Rab31 expression inhibited BT-549 cell proliferation, while Rab31 overexpression promoted MDA-MB-231 cell proliferation (Figure 5F). Wound healing assays revealed reduced migration in Rab31-knockdown BT-549 cells compared to control at 24 and 48 h, and increased migration in Rab31-overexpressing MDA-MB-231 cells at 24 and 48 h (Figure 5G). Transwell assay demonstrated fewer BT-549 cells passing through the filter membrane after Rab31 knockdown, and more MDA-MB-231 cells passing through upon Rab31 overexpression (Figure 5H). These results suggest that Rab31 knockdown inhibits migration and invasion in BT-549 cells, while Rab31 overexpression enhances these capabilities in MDA-MB-231 cells.

Additionally, we examined the role of Rab31 in autophagy. Results showed that interfering with Rab31 expression suppressed autophagy-related proteins, while Rab31 overexpression enhanced them (Figure 5I and Figure S1). This suggests Rab31 plays a key role in the autophagy signaling pathway and promotes triple-negative breast cancer progression.

### 3.6. Knockdown of Rab31 Reversed the Effects of Cx43 Overexpression on Autophagy and Biological Characteristics in Triple-Negative Breast Cancer Cells

**Figure 6:**
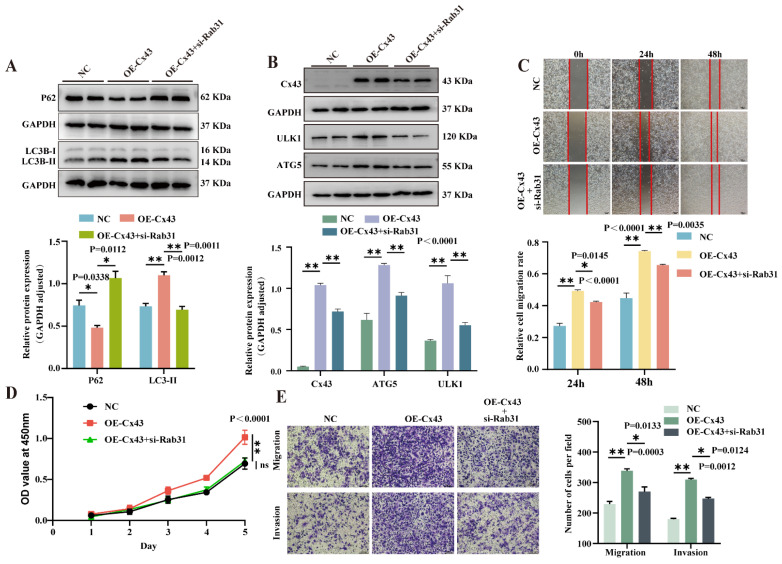
Cx43 regulates the autophagy pathway through Rab31 in MAD-MB-231. (**A**,**B**) WB analysis of Cx43 and key autophagy markers (P62, LC3, ATG5, ULK1) in Cx43-overexpressing breast cancer cells (MDA-MB-231) following Rab31 modulation. (**C**) A wound healing assay was employed to assess cell migration and migration rate. (**D**) The CCK8 assay was employed to evaluate the cell proliferation (statistical significance was determined using two-way ANOVA). (**E**) Transwell assay was employed to evaluate cell migration and invasion (n = 3 independent experiments (biological replicates). Statistical significance was determined using one-way ANOVA with Tukey’s post hoc test. Mean ± SD, * *p* < 0.05, ** *p* < 0.01, ns, not significant (*p *> 0.05).). Original western blots are presented in File S1.

To further investigate the role of Cx43 binding to Rab31 in autophagy and breast cancer cell biology, we knocked down Rab31 expression in MDA-MB-231 cells overexpressing Cx43. Western blot revealed a significant reduction in Cx43 levels in OE-Cx43 cells transfected with si-Rab31, as compared to OE-Cx43 cells. Knockdown of Rab31 reversed the increased expression of Cx43 and autophagy-related proteins ULK1, ATG5, LC3, and p62 (Figure 6A,B and Figure S1). CCK-8 assay revealed inhibited proliferation in OE-Cx43 cells transfected with si-Rab31 compared to OE-Cx43 cells alone. Wound healing demonstrated reduced migration ability, while the transwell assay showed fewer migrating and invading cells in OE-Cx43 cells transfected with si-Rab31, indicating suppressed migration and invasion capabilities (Figure 6C–E). These results suggest that Cx43 promotes breast cancer progression via the Rab31/ULK1/autophagy pathway.

### 3.7. Overexpression of Cx43 Promotes Triple-Negative Breast Tumor Growth In Vivo

**Figure 7:**
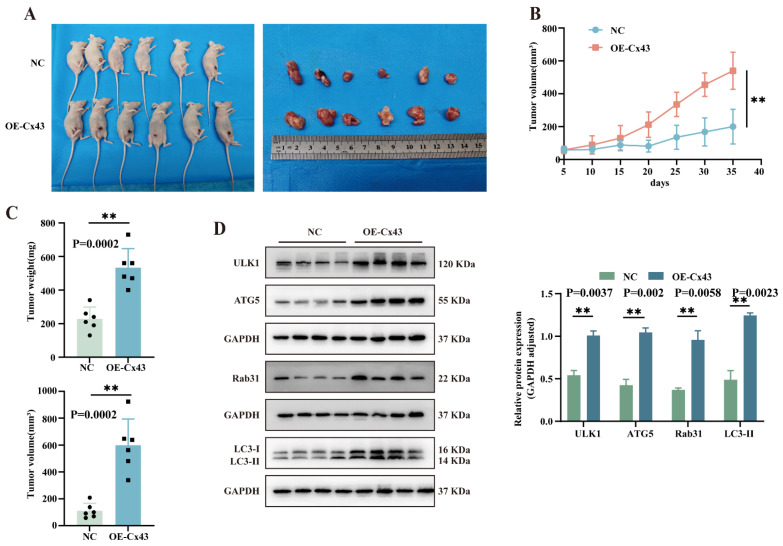
Overexpression of Cx43 promotes breast tumor growth in vivo. (**A**) Representative images of orthotopic transplanted tumors in the control and Cx43 overexpression groups. (**B**) Tumor growth in vivo was assessed every five days and the growth curves were plotted (statistical significance was determined using two-way ANOVA). (**C**) Animals were sacrificed to remove tumors, and the weight and volume of tumors were determined. (**D**) The expression of Rab31 and autophagy-related proteins in tumor tissues was assessed using WB (n = 6 independent tumor samples per group; statistical significance was determined using an unpaired *t*-test, mean ± SD, ** *p* < 0.01). Original western blots are presented in File S1.

An orthotopic nude mouse model of breast cancer was used to study the effect of Cx43 on tumor growth in vivo. MDA-MB-231 cells stably overexpressing Cx43 (OE-Cx43) and control cells (NC) were injected into female nude mice mammary glands. Results showed that Cx43 overexpression significantly increased tumor growth rate, volume, and size (Figure 7A–C). Western blot revealed high levels of Rab31, ULK1, ATG5, and LC3 following Cx43 overexpression (Figure 7D). These findings support the in vitro observations, suggesting that Cx43 promotes triple-negative breast cancer progression by activating autophagy via Rab31 regulation.

## 4. Discussion

Breast cancer represents a highly heterogeneous malignant tumor prevalent among women, posing a significant threat to their health. Various treatment regimens have been developed in response to the distinct molecular subtypes of breast cancer. However, TNBC, characterized by the absence of ER, PR, and HER2, presents a unique challenge. Due to the lack of these receptors, therapeutic options for TNBC are notably limited, leading to reduced survival times. In particular, metastatic TNBC has an even poorer prognosis, with a 5-year survival rate ranging from 10 to 20% [28]. Therefore, there is an urgent need to explore new therapeutic targets and strategies to improve patient outcomes.

Cx43 is a predominant subtype within the family of gap junction proteins, facilitating intercellular communication and substance exchange via paracrine signaling [29]. The expression and localization of Cx43 are intricately associated with cellular functions. Previous studies have demonstrated that Cx43 can serve as a protective factor, inhibiting tumor growth [30,31,32]. Kazan et al. [32] found Cx43 underexpressed in TNBC, functioning as a tumor suppressor that maintains the epithelial phenotype and inhibits EMT/metastasis. Fu et al. [33] showed that Cx43 downregulates EMT by inhibiting the TGFβ1-Smad3-ITGαV axis, suppressing metastasis. Meanwhile, McLachlan et al. [34] and Hirrschi et al. [35] showed that Cx43 overexpression inhibits malignant phenotypes in primary tumors via non-channel-dependent mechanisms, independent of functional gap junctions. Consequently, Cx43 is widely regarded as a tumor suppressor in breast cancer, as reviewed by Grek et al. [36]. However, recent findings indicate that Cx43 may also promote tumor progression. Tishchenko et al. [37] indicated that Cx43 promotes the formation of tunneling nanotubes (TNTs). As direct intercellular channels, TNTs are critically involved in cancer cell communication, metastasis, and the development of drug resistance. This finding positions Cx43 as a factor contributing to tumor progression and metastasis. Furthermore, Shen et al. [38] demonstrated that Cx43 contained within exosomes serves as a potential biomarker, also implicating its non-canonical functions in extracellular communication and the remote regulation of the tumor microenvironment. This exosomal Cx43 is suggested to facilitate the formation of the pre-metastatic niche, thereby promoting metastatic dissemination. Zibara et al. [39] found that functional Cx43 channels promote transendothelial migration during metastasis via tumor-endothelial communication, which can be blocked by gap junction inhibitors, potentially due to a variety of factors, such as differences in model systems, differences in tumor development stage, specificity of microenvironment, changes in subcellular localization of Cx43, and post-translational modifications. In this study, we examined Cx43 expression in triple-negative breast cancer tissues using immunohistochemistry and observed significantly higher Cx43 expression in cancerous tissues compared to adjacent non-cancerous tissues. Additionally, at the cellular level, two triple-negative breast cancer cell lines (MDA-MB-231 and BT-549) exhibited markedly elevated mRNA expression levels of Cx43 compared to normal breast epithelial cells (MCF-10A) as determined using quantitative PCR. Furthermore, we quantified Cx43 protein levels in two TNBC cell lines using Western blot analysis. Our results demonstrated that Cx43 was significantly upregulated in these TNBC cells compared to negligible expression in normal breast epithelial cells. A comprehensive series of functional assays, including CCK8 proliferation assays, wound healing assays, and transwell migration and invasion assays, revealed that inhibition of Cx43 expression markedly reduced cellular proliferation, migration, and invasion capabilities. Conversely, overexpression of Cx43 enhanced these malignant phenotypes. These findings suggest a critical role for Cx43 in the pathogenesis of breast cancer, particularly in TNBC, indicating its potential significance in tumor progression.

As a transmembrane protein, the carboxyl terminus of Cx43 can interact with various kinases and cytoplasmic proteins, functioning as a signaling hub [40,41]. In this study, we utilized gene and protein interaction databases to predict potential interacting partners of Cx43. Our analysis revealed that Cx43 and Rab31 exhibit similar expression patterns and cellular localization. Rab proteins, members of the GTP-binding protein family, are approximately 200 amino acids in length, characterized by a conserved G domain and highly variable N- and C-termini. These proteins primarily localize to the trans-Golgi network and endosomes, where they regulate intracellular membrane trafficking and vesicle transport. Rab31, a member of the Rab family of small GTP-binding proteins, plays a crucial role in membrane transport regulation within eukaryotic cells [42]. Immunofluorescence assays demonstrated co-expression of Cx43 and Rab31 in the cytoplasm, while co-immunoprecipitation experiments confirmed their physical interaction. Furthermore, the knockdown of Cx43 expression resulted in decreased Rab31 protein levels, whereas Cx43 overexpression led to increased Rab31 expression. This suggests a positive correlation between Cx43 and Rab31 expression, indicating that Cx43 may exert regulatory effects on Rab31.

Rab31 is a critical Rab GTPase that plays a pivotal role in cellular membrane trafficking and vesicle formation. In line with previous reports of Rab31 overexpression in multiple malignancies [43,44,45,46,47], we also detected its expression in TNBC cell lines. Consequently, we evaluated the expression levels of Rab31 using qPCR and Western blotting in breast epithelial cells (MCF-10A) and two triple-negative breast cancer cell lines (MDA-MB-231 and BT-549). Our results revealed significantly higher expression levels of Rab31 in the triple-negative breast cancer cells compared to the breast epithelial cells, with statistically significant differences.

To further investigate the impact of Rab31 on the biological characteristics of breast cancer cells, we conducted a series of experiments involving Rab31 knockdown and overexpression. Using CCK8 assays, wound healing assays, and transwell migration and invasion assays, we observed that inhibition of Rab31 expression led to reduced cell proliferation, migration, and invasion. Conversely, overexpression of Rab31 enhanced these cellular activities. Research has indicated that Rab31 may activate autophagy through the PI3K/AKT/mTOR signaling pathway by accelerating the degradation rate of ligand-bound EGFR. However, the precise mechanisms underlying the role of Rab31 in breast cancer remain to be elucidated. Additionally, it has not been reported whether Cx43 can induce cellular energy changes by binding to Rab31, thereby activating autophagy.

Autophagy is a cellular process that involves the degradation and recycling of intracellular components. It serves to maintain cellular metabolism and survival under conditions of starvation and stress, while also eliminating damaged proteins and organelles to ensure the quality and quantity of these cellular components [48]. Autophagy plays a significant pathophysiological role in various disease processes [49,50] and exhibits a dual function during different stages and contexts of tumor development [51]. Research has demonstrated that autophagy is a critical factor for the survival of numerous cancer cells [52]. In advanced stages of tumorigenesis, autophagy can enhance tumor survival and growth by scavenging toxic oxygen-free radicals, maintaining mitochondrial function, sustaining metabolism and survival under stress, and preventing tumor cell damage [53,54]. Additionally, multiple studies have established a link between increased autophagy and metastasis in several types of cancer, including breast cancer [55], melanoma [56], hepatocellular carcinoma [57], and glioblastoma [58], suggesting that autophagy can promote cancer metastasis and increase the invasiveness of cancer cells.

In this study, we observed that the inhibition of Cx43 expression led to a decrease in ULK1 expression, whereas the overexpression of Cx43 increased ULK1 expression. Notably, ULK1 is a serine/threonine kinase whose loss can disrupt autophagy in various cell lines. As a critical component in the initiation of autophagy, ULK1 plays a pivotal role in this process [59,60]. Additionally, we examined the protein expression levels of other autophagy-related proteins, including p62, LC3B, and ATG5, and found consistent results: the expression levels of ATG5 and LC3B mirrored those of Cx43, while p62 expression exhibited an inverse trend. These findings further suggest that Cx43 may influence breast cancer development by modulating ULK1-mediated autophagy. Furthermore, after interfering with or overexpressing Rab31, we detected changes in the expression levels of autophagy-related proteins such as p62, LC3B, ULK1, and ATG5. We found that inhibiting Rab31 expression suppressed autophagy, while enhancing Rab31 expression activated it. Collectively, these experiments indicate that Rab31 may promote the progression of triple-negative breast cancer by influencing key proteins in the autophagy pathway.

These results raise an important question regarding the apparent contradiction between our findings and established knowledge of Cx43’s role in autophagy regulation. Previous studies by Bejarano et al. [61] established that Cx43 normally suppresses autophagy through interactions with Atg16 and PI3K complexes, with this inhibition being reversible upon starvation-induced internalization. Tittarelli et al. [62] revealed in melanoma that hypoxia-induced autophagy selectively degrades gap junctional Cx43 at the immunological synapse, destabilizing the NK-tumor cell interface. This impairs immune-mediated killing and promotes tumor immune escape. Murphy SF et al. [63] demonstrated in glioblastoma that inhibiting Cx43 via the αCT1 peptide blocks the AKT/mTOR pathway, thereby activating autophagy. This synergizes with temozolomide to induce autophagy-dependent cell death and reverse chemotherapy resistance. The above indicates that autophagy–Cx43 regulation exhibits duality: inhibition of Cx43 triggers pro-death autophagy, whereas selective autophagy degrades functional Cx43 to enable immune escape. However, our current findings demonstrate a paradoxical pro-autophagic role of Cx43 in TNBC, where it upregulates Rab31 to enhance autophagy and promote tumor progression. This functional reversal likely stems from three key factors: (1) the unique metabolic stresses (hypoxia, nutrient deprivation) of the TNBC microenvironment that may repurpose Cx43 to support tumor survival. (2) preferential interaction with Rab31 rather than Atg16, activating autophagy through the Rab31-ULK1 axis. (3) tumor-specific post-translational modifications (e.g., Src-mediated phosphorylation) that alter Cx43 binding properties. These findings illustrate the context-dependent duality of Cx43 function—maintaining homeostasis in normal physiology while potentially driving malignancy in tumors through pathway-specific adaptations to cellular stress.

An in-depth investigation into the molecular mechanisms and regulatory roles of Cx43 in TNBC was designed through reversion experiments. Specifically, the knockdown of Rab31 expression in Cx43-overexpressing cells significantly suppressed the expression of autophagy-related proteins and attenuated the phenotypic characteristics associated with breast cancer. This indicates that inhibiting Rab31 expression effectively reverses the effects of Cx43 overexpression, which otherwise activates autophagy and promotes breast cancer cell proliferation, migration, and invasion. These findings suggest that Cx43 may facilitate breast cancer progression by interacting with Rab31 and activating autophagy. Our results align with previous studies on other Rab proteins in cancer contexts. Data from our study indicate that Cx43 overexpression may enhance tumor growth. In tumor tissues, Cx43 overexpression upregulates Rab31 expression, and various assays were employed to examine downstream molecules regulated by Cx43. The expression levels of ULK1, p62, LC3B, and ATG5 were also modulated. These observations corroborate our in vitro findings. Collectively, we hypothesize that Cx43 promotes distant metastasis of breast cancer by influencing autophagy via Rab31, potentially offering novel insights and therapeutic targets for clinical breast cancer research and treatment. Additionally, inhibiting Cx43 could represent a new approach to treating breast cancer.

## 5. Conclusions

In summary, our findings indicate that Cx43 is significantly overexpressed in TNBC. We have demonstrated that Cx43 promotes the proliferation, migration, and invasion of breast cancer cells in vitro. Additionally, we verified that Cx43 overexpression enhances the growth of breast cancer cells in vivo using a murine model. Mechanistically, our data reveal that Cx43 overexpression markedly upregulates Rab31 expression and stimulates autophagy activity via ULK1 activation, leading to increased LC3B levels and decreased p62 levels. These results suggest that Cx43 plays a pivotal role in regulating autophagy in breast cancer cells. Specifically, Cx43 may directly or indirectly modulate Rab31 function, thereby influencing the formation and degradation processes of autophagosomes. This regulatory effect is likely closely associated with Cx43’s role as a connexin in intercellular communication and signaling. Consequently, Cx43 holds potential as a novel therapeutic target for TNBC patients. Certainly, this study acknowledges several limitations. The expression of Cx43 in breast cancer remains a topic of debate, and the relationship between Cx43 and various subtypes of breast cancer, as well as lymph node metastasis, requires further investigation. While our preliminary IHC analysis of five samples suggested higher Cx43 expression in TNBC compared to normal tissues, this finding necessitates rigorous validation in a larger, statistically powered cohort. Additionally, although we identified that Cx43 and Rab31 synergistically regulate autophagy, whether they act through identical signaling pathways remains to be determined. The precise mechanism by which Rab31 participates in the modulation of autophagy signaling pathways remains to be explored. Future studies should employ more rigorous autophagic flux assays to elucidate these issues. Finally, our study has identified that Cx43 is highly expressed in triple-negative breast cancer cells and may activate autophagy-related pathways through the regulation of Rab31, thereby influencing the progression of breast cancer. However, the role of Cx43 in other subtypes of breast cancer cells warrants additional research to fully elucidate its regulatory function. Addressing these gaps will guide our future research endeavors.

## Figures and Tables

**Table 1:** The primer sequences for RT-qPCR.

		F (5′→3′)	R (5′→3′)
Cx43		AGTTCAATCACTTGGCGTGACTTC	GTTTGCCTAAGGCGCTCCAG
Rab31		GACCACAACATCAGCCCTACTAT	GCCAATGAATGAAACCGTTCCT
GAPDH		GCACCGTCAAGGCTGAGAAC	TGGTGAAGACGCCAGTGGA
si-NC		UUCUCCGAACGUGUCACGUTT	ACGUGACACGUUCGGAGAATT
si-Cx43	#1	UGAAGCAGAUUGAGAUAAATT	UUUAUCUCAAUCUGCUUCATT
	#2	CAAGCAAGCAAGUGAGCAATT	UUGCUCACUUGCUUGCUUGTT
	#3	CUGAUGACCUGGAGAUCUATT	UAGAUCUCCAGGUCAUCAGTT
si-Rab31	#1	UGAAGGAUGCUAAGGAAUATT	UAUUCCUUAGCAUCCUUCATT
	#2	GGACACUGGGGUUGGGAAATT	UUUCCCAACCCCAGUGUCCTT
	#3	GGUUGAGACAAGUGCAAAATT	UUUUGCACUUGUCUCAACCTT

## Data Availability

The datasets used and/or analyzed during the current study are available from the first author upon reasonable request.

## Supplementary Materials

The following supporting information can be downloaded at: https://www.mdpi.com/article/10.3390/cancers17243923/s1, Supplementary Figure S1. Quantitative analysis of LC3-II/I ratios from the original study. (A) Data corresponding to Figure 4E. (B) Data corresponding to Figure 5I. (C) Data corresponding to Figure 6A. Data are from n = 3 independent experiments (biological replicates) and are presented as mean ± SD. Statistical significance was determined by an unpaired t-test (* *p* < 0.05, ** *p* < 0.01). File S1: Original western blots.

## List of Abbreviations

Abbreviation	Full Term
TNBC	Triple-negative breast cancer
Cx43	Connexin43
Rab31	Ras-related protein Rab-31
ULK1	Unc-51-like autophagy activating kinase 1
ATG5	Autophagy-related gene 5
LC3	Microtubule-associated proteins 1A/1B light chain 3
SQSTM1/p62	Sequestosome 1
CTC	Circulating tumor cell
OE-CX43/NC	Over-expression of CX43/ negative control
si-CX43/si-NC	siRNA targeting CX43/interference negative control
OE-Rab31/vector	Over-expression of RAB31/negative control
GJ	Gap junction
ER	Estrogen receptor
PR	Progesterone receptor
HER2	Human epidermal growth factor
WB	Western blot
IHC	Immunohistochemistry
