# Supplementary material for: The Cx43-Mediated Autophagy Mechanism Influences Triple-Negative Breast Cancer Through the Regulation of Rab31
Source: Cancers (Basel). 2025 Dec 8;17(24):3923. doi: 10.3390/cancers17243923 (PMC12730575; doi:10.3390/cancers17243923)
Supplement: Supplementary file 1 [file cancers-17-03923-s001.zip › Supplementary Figure S1.pdf]

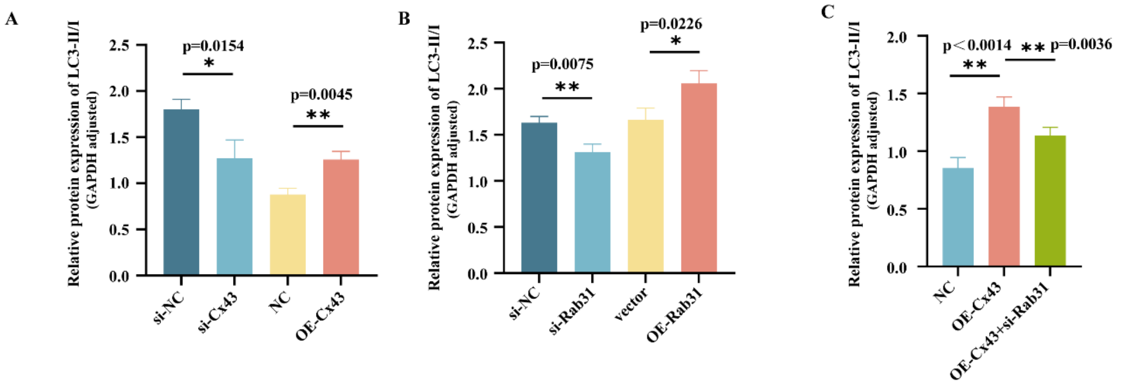

Figure S1. **Quantitative analysis of LC3-II/I ratios from the original study. A)** Data corresponding to Fig. 4E. **B)** Data corresponding to Fig. 5I. **C)** Data corresponding to Fig. 6A. Data are from n = 3 independent experiments (biological replicates) and are presented as mean ± SD. Statistical significance was determined by an unpaired t-test (*$P < 0.05$, **$P < 0.01$).
