# Supplementary figures and images for: The Cx43-Mediated Autophagy Mechanism Influences Triple-Negative Breast Cancer Through the Regulation of Rab31
Source: Cancers (Basel). 2025 Dec 8;17(24):3923. doi: 10.3390/cancers17243923 (PMC12730575; doi:10.3390/cancers17243923)

**Fig.1D**

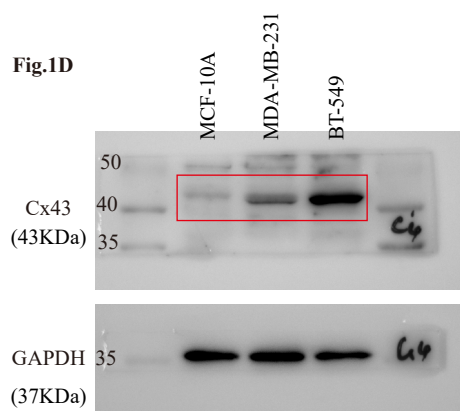

**Fig.2B**

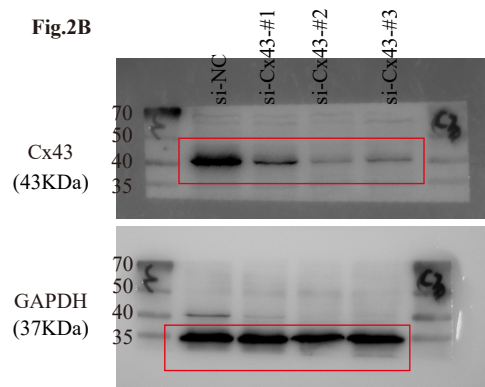

**Fig.2D**

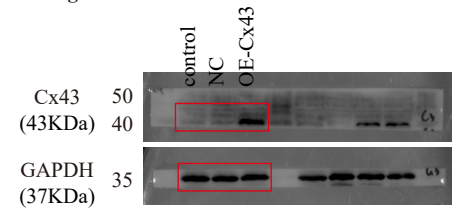

**Fig.4C**

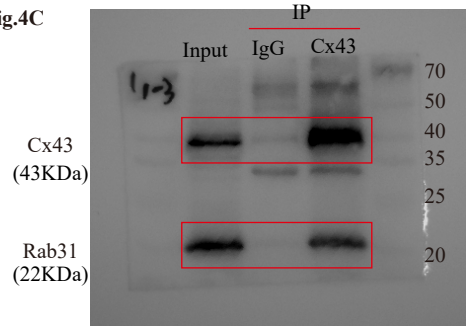

**Fig.4D**

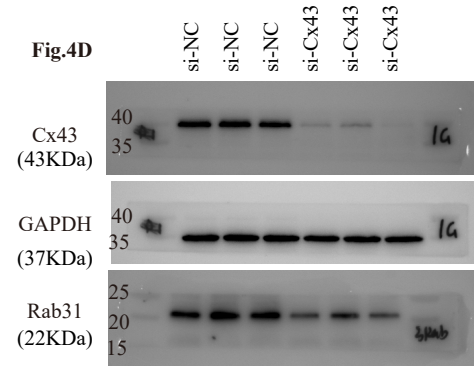

**Fig.4E**

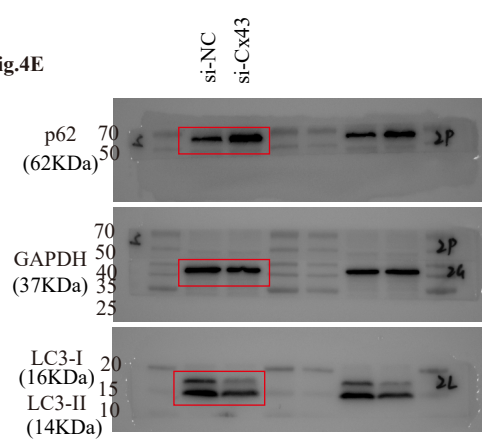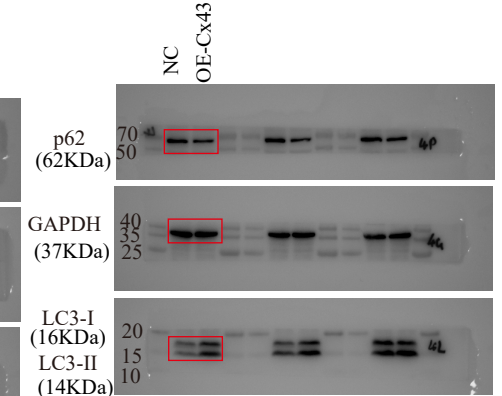

**Fig.4F**

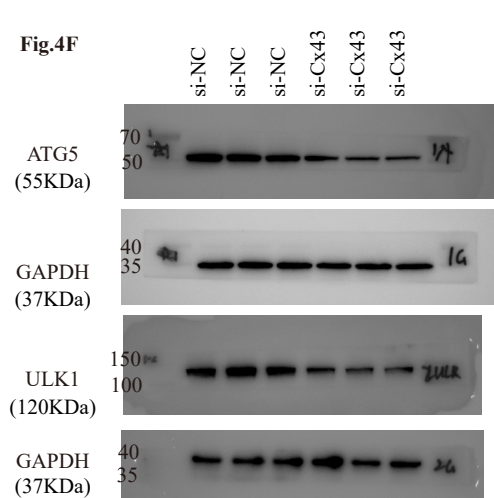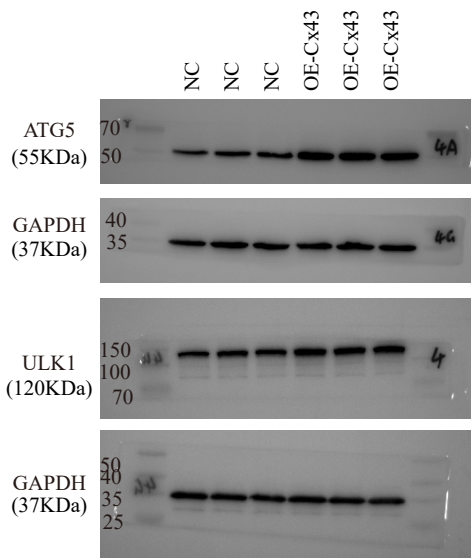

Fig.5A

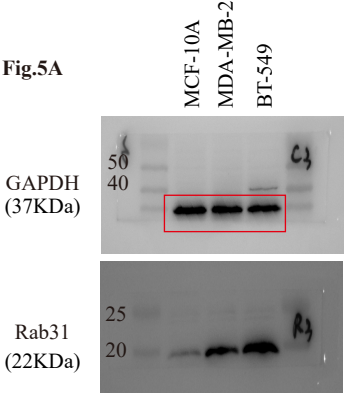

Fig.5C

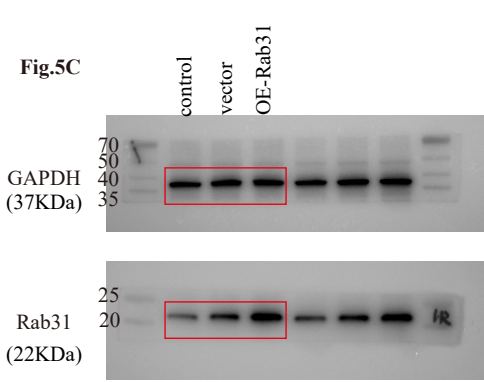

Fig.5E

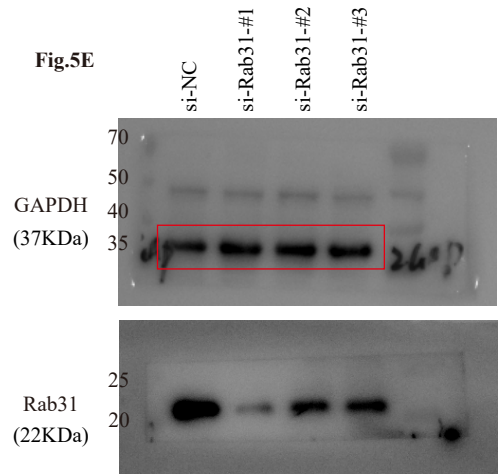

Fig.5I

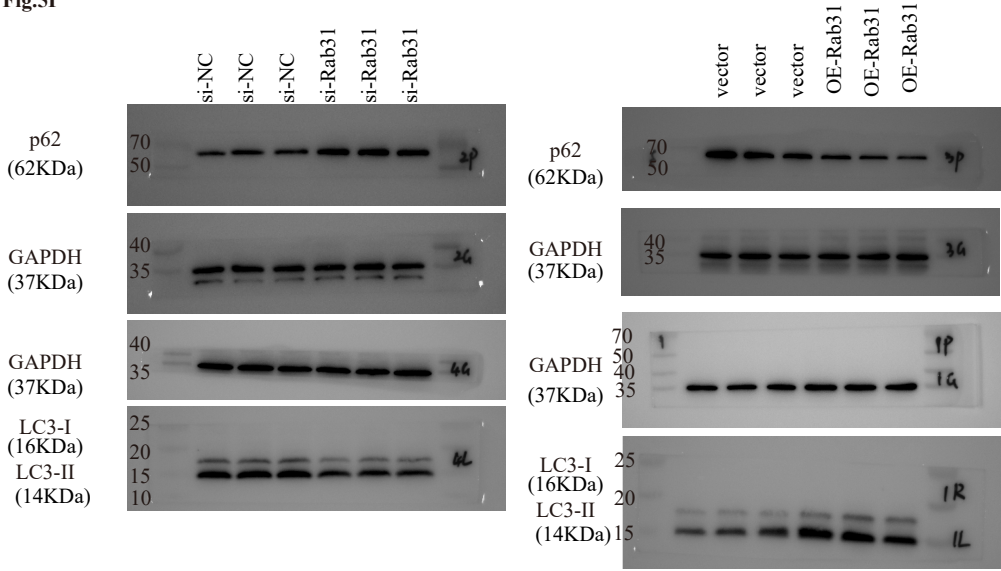

Fig.6A

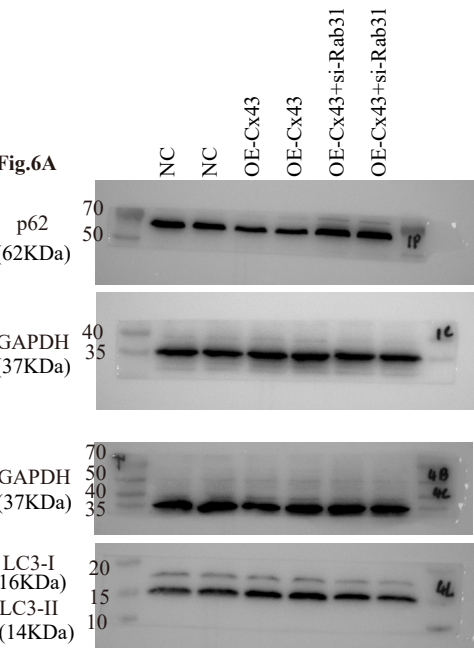

Fig.6B

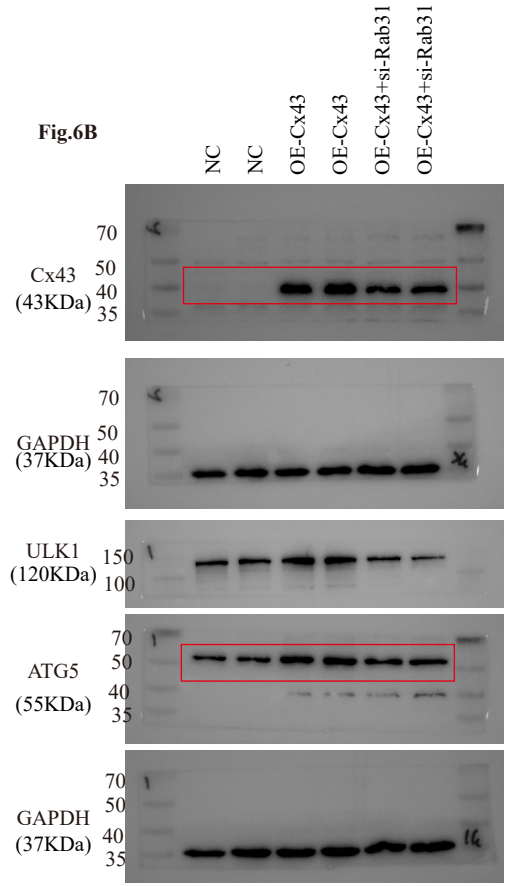

Fig.7D

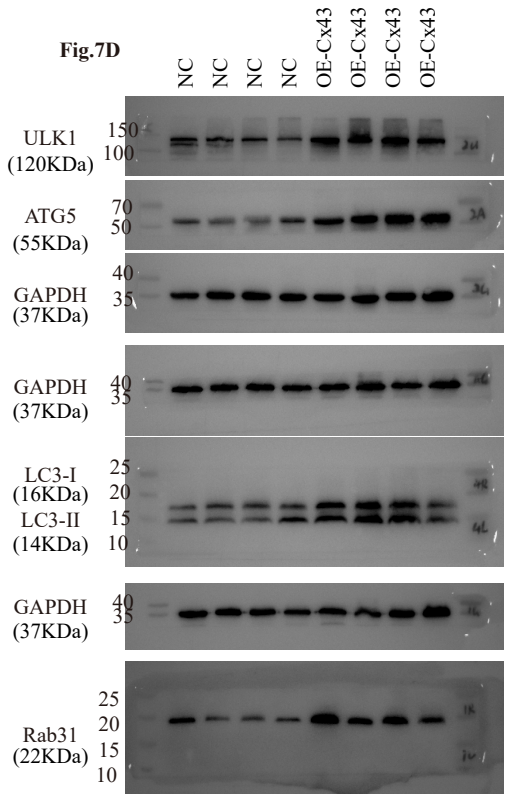

Supplement: Supplementary file 1 [file cancers-17-03923-s001.zip › File S1.pdf]
